# Triggering mitophagy with far-red fluorescent photosensitizers

**DOI:** 10.1038/srep10376

**Published:** 2015-05-29

**Authors:** Cheng-Wei Hsieh, Chih-Hang Chu, Hsien-Ming Lee, Wei Yuan Yang

**Affiliations:** 1 Chemical Biology and Molecular Biophysics Program, Taiwan International Graduate Program; 2 Institute of Biological Chemistry; 3 Institute of Chemistry, Academia Sinica, Taipei, 115 Taiwan; 4 Institute of Biochemical Sciences, College of Life Sciences, National Taiwan University, Taipei 106, Taiwan

## Abstract

Cells identify defective mitochondria and eliminate them through mitophagy: this allows cells to rid themselves of unwanted stress to maintain health and avoid the activation of cell death. One approach to experimentally investigate mitophagy is through the use of mitochondrial photosensitizers, which when coupled with light allows one to precisely control mitochondrial damage with spatial and temporal precision. Here we report three far-red fluorophores that can be used as robust mitochondrial photosensitizers to initiate mitophagy. The dyes offer maximal compatibility with multi-color live-cell imaging, as they do not spectrally overlap with commonly used fluorescent proteins. Through the use of these far-red fluorescent photosensitizers we found that mitophagic engulfment and mitophagosome maturation rates are highly correlated with the cellular Parkin-labeled mitochondria levels. This may represent a protective cellular mechanism to avoid membrane and lysosome depletion during mitophagy.

Cells maintain a functional mitochondrial network and avoid cellular buildup of defective mitochondria through proper mitochondrial damage responses. These responses include mitochondrial biogenesis, proteolytic removal of defective mitochondrial proteins, selective exportation of defective mitochondrial components through vesicular trafficking, and wholesale mitochondrial degradation through autophagy^1^,^2^,^3^,^4^,^5^. Defective mitochondrial quality control can lead to unwanted cell stress or even the activation of cell death.

Experimental strategies that specifically elicit mitochondrial injury allow robust probing of mitochondrial quality control pathways. Mitochondrial injury can be induced through the administration of small molecules (such as those that act as mitochondrial toxins) or the overexpression of mitochondrial targeted, aggregation-prone proteins^4^,^6^,^7^. For example these approaches allowed identification of PINK1/Parkin in the maintenance of mitochondria. It was found that the ubiquitin E3 ligase Parkin selectively translocate onto dysfunctional mitochondria to mediate their selective turnover through autophagy. This process, often termed Parkin-mediated mitophagy, is now one of the best characterized mitochondrial maintenance pathways, and is implicated in Parkinson’s disease^8^,^9^,^10^,^11^,^12^.

A recently developed strategy that specifically elicits mitochondrial injury involves the use of mitochondria-targeted photosensitizers^13^,^14^,^15^. These efforts utilized either the genetically-encoded red fluorescent protein KillerRed or the mitochondria membrane-potential sensitive dye tetramethylrhodamine (TMRE) to achieve light-activated mitochondrial damage. Irradiation of KillerRed- or TMRE-containing mitochondria by green light leads to the generation of reactive oxygen species (ROS) within the mitochondrial matrix, resulting in selective oxidation of mitochondrial components. This was shown to be sufficient to trigger Parkin-mediated mitophagy. The advantage light-activation schemes offer is precise temporal control of mitochondrial damage with subcellular resolution. This, for example, allows one to selectively impair mitochondria in distal axons for investigating mitophagy in neurons^16^. Additionally the amount of mitochondria to be impaired can be precisely controlled through illumination, adding flexibility for investigating mitochondrial quality control pathways.

Here we report the identification of far-red fluorescent photosensitizers that can be utilized to robustly activate mitophagy. These photosensitizers do not spectrally overlap with blue, green, or red fluorescent proteins and are therefore more compatible with multi-color live-cell imaging. We carefully characterized three commercially available far-red photosensitizers that label mitochondria. All three exclusively localized to mitochondria and specifically elicited mitophagy with subcellular resolution when irradiated with low levels of 635 nm light. Among the three dyes Mitotracker DeepRed FM is most optimal when combined use of light-activated mitophagy and three-color live-cell imaging is desired. We took advantage of the newly identified photosensitizers to monitor mitophagy progression under varying amount of substrates. We find that mitophagy efficiency is strongly influenced by substrate level, and this is reflected at both the autophagic engulfment and mitophagosome maturation steps.

## Results

### Robust far-red fluorescent photosensitizers that targets mitochondria

**Figure 1:**
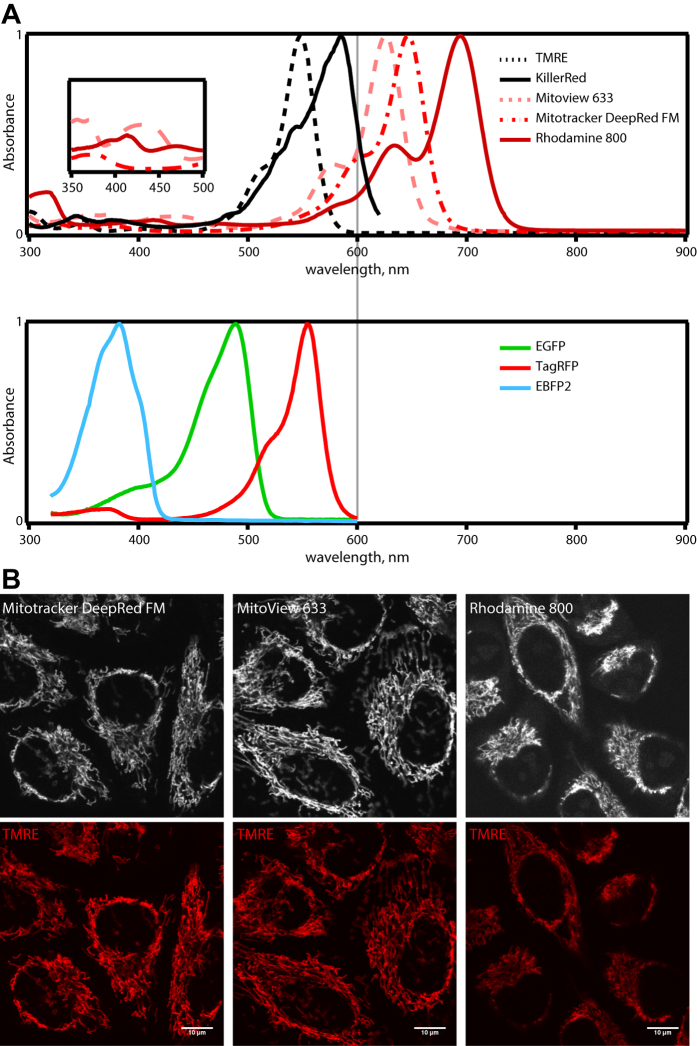
Spectral and staining properties for Mitotracker Deep Red FM, Mitoview 633, and Rhodamine 800. (**A**) *Top row:* Absorbance spectra for individual far-red fluorescent photosensitizers in PBS, overlayed with those for TMRE and KillerRed. *Inset:* far-red fluorescent photosensitozers’ absorbance in the 350–500 nm range. *Bottom row:* Absorbance spectrum for EBFP2, EGFP, and TagRFP. Peaks of each absorbance spectrum were normalized to 1. (**B**) *Top row:* Staining patterns for Mitotracker Deep Red FM, Mitoview 633, and Rhodamine 800 in HeLa cells. *Bottom row:* HeLa cell mitochondrial network visualized through TMRE. All three photosensitizers display highly selective labeling of mitochondria in HeLa cells. Scale bars, 10 μm.

Previous schemes to light-activate Parkin-mediated mitophagy relied on the use of red fluorescent photosensitizers KillerRed or TMRE^13^,^14^,^15^. These two dyes, however, spectrally overlap with red fluorescent proteins (Fig. 1A). This limits their combined use with multi-color live-cell imaging (as they leave only the blue and green emission channels open). To overcome this limitation it is therefore desirable to identify far-red or infrared fluorescent photosensitizers capable of eliciting mitochondrial damage.

We therefore carefully examined whether three commercially available far-red fluorescent mitochondrial probes, Mitotracker DeepRed FM, Mitoview 633, and Rhodamine 800^17^ can be used for triggering mitophagy . As shown in Fig. 1A, these three dyes are spectrally red-shifted from commonly used probes for live-cell imaging, such as EBFP2, EGFP, and TagRFP (therefore compatible with 3-color live cell imaging). Their staining patterns in HeLa cells revealed highly selective labeling of the entire mitochondrial network (Fig. 1B) - this specificity is required to afford selective ROS generation within mitochondria upon light illumination. Their staining also did not perturb the mitochondrial membrane potential (Fig. 1B; no loss in tetramethylrhodamine ethyl ester (TMRE) flourescence). Their use require red excitation (e.g. 635 nm), a color that does not by itself disrupt intracellular organelles when used at sub-mW intensities^18^.

**Figure 2:**
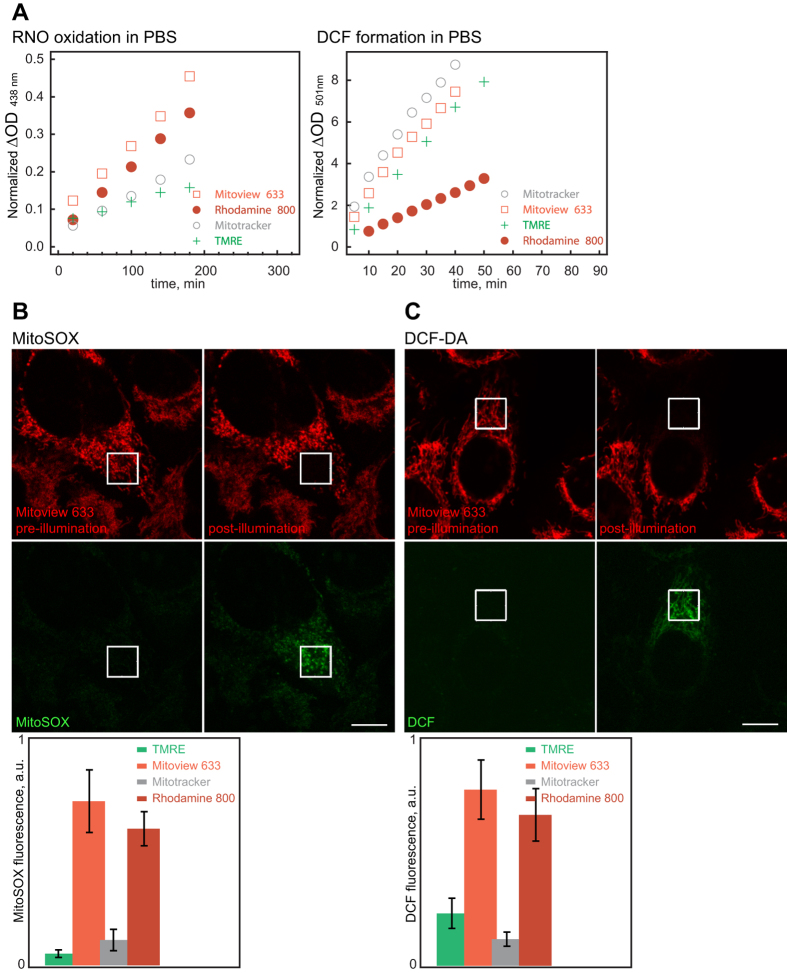
ROS production ability comparison between Mitotracker Deep Red FM, Mitoview 633, Rhodamine 800, and TMRE. (**A**) Far-red fluorescent photosensitizers or TMRE were continuously illuminated *in vitro* (in PBS) and assayed for ROS generation through RNO oxidation (*left panel*) or DCF formation (*right panel*). All three far-red fluorescent photosensitizers displayed the same order of magnitude ROS production ability as TMRE in PBS (Materials and Methods). (**B**) HeLa cells stained with 100 nM photosensitizers were illuminated within 8 μm × 8 μm regions and assayed for mitochondrial ROS production through mitoSOX. *Images:* Example using Mitoview 633. 635 nm illumination within the white square elicited spatially restricted increase in mitoSOX fluorescence (*left:* before illumination; *right:* after illumination). *Bottom graph:* relative mitoSOX fluorescence increase within the illuminated region for different photosensitizers. (**C**) Same as in **B** but assayed through DCF generation. 635 nm illumination led to spatial restricted DCF formation within mitochondria. Scale bars, 10 μm.

Next we asked whether Mitotracker DeepRed FM, Mitoview 633, and Rhodamine 800 are strong enough photosensitizers for inducing mitochondrial dysfunction. We quantified light-activated ROS production for the three far-red fluorescent dyes both in aqueous solution as well as directly within the HeLa cell mitochondrial matrix. Their ability to mediate ROS production in both of these environments was benchmarked against that of TMRE, as previous work has determined that TMRE is a robust photosensitizer for triggering mitochondrial dysfunction and Parkin-mediated mitophagy^13^. Two assays were used for measuring ROS production in solution: (**a**) N,N-Dimethyl-4-nitrosoaniline (RNO) plus imidazole that detects singlet oxygen formation^19^, and (**b**) general ROS detection through dichlorofluorescein (DCF) formation^20^ (Materials and Methods). The three far-red fluorophores elicited the same order of magnitude responses as TMRE in the two *in vitro* assays (Fig. 2A). On the other hand ROS production within the HeLa cell mitochondrial matrix were measured using mitoSOX and 2’, 7’-dichlorofluorescin diacetate (DCF-DA). MitoSOX is a live-cell permeant probe that selectively targets mitochondria and exhibits bright red fluorescence when oxidized by superoxides^21^. DCF-DA is a general ROS sensor that, when oxidized to DCF, becomes highly green-fluorescent^22^. Compared to TMRE the far-red dyes (using 100 nM staining conditions) elicited either similar or stronger mitoSOX or DCF fluorescence within mitochondria when irradiated (Fig. 2B; without dye-staining, 635 nm illumination did not elicit detectable changes in DCF or mitoSOX fluorescence in HeLa cells). Together these results strongly suggested that Mitotracker DeepRed FM, Mitoview 633, or Rhodamine 800 will all be adequate for mediating mitochondrial damage.

### Live-cell imaging with mitophagy-triggering far-red photosensitizers

**Figure 3:**
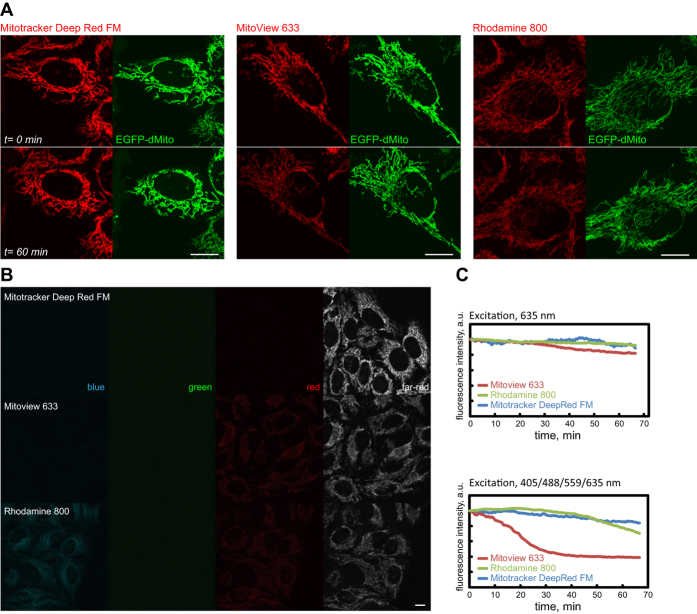
Compatibility with live-cell imaging. (**A**) Photosensitizer-stained HeLa cells expressing EGFP-dMito were imaged with 4 colors (405 nm, 488 nm, 559 nm, 635 nm) at 1 min intervals for 61 frames. The presence of photosensitizers did not affect cell health or viability, evidenced by the unaltered cell morphologies and tubular mitochondria after completion of time-lapse imaging. (**B**) Photosensitizer-stained HeLa cells’ fluorescence in blue (405 nm excitation, 430–470 nm emission), green (488 nm excitation, 505–540 nm emission), red (559 nm excitation, 575–620 nm emission) and far-red channels (635 nm excitation, 655–755 nm emission). (**C**) Photostability of far-red fluorescent photosensitizers under 635 nm or simultaneous 405/488/559/635 nm illumination conditions. Scale bars, 10 μm.

Additionally we addressed the dyes’ suitability for long term multi-color live cell imaging. For this the far-red dyes should ideally fluoresce minimally in blue, green, and red, and display low cytotoxicity. HeLa cells (expressing EGFP-dMito to highlight the mitochondrial network) stained with 100 nM Mitotracker DeepRed FM, Mitoview 633, or Rhodamine 800 were imaged with four colors (405, 488, 559, and 635 nm). While the far-red dyes are robust photosensitizers, their presence during live-cell imaging did not lead to apparent mitochondrial damage or cell stress, as evidenced by the lack of mitochondrial fragmentation, cell deformation, or cell death during the entire imaging time span (Fig. 3A). Among the far-red dyes Mitotracker Deep Red FM displayed the least amount of crosstalk into the blue, green, or red emission channels (Fig. 3B). Mitoview 633 and Rhodamine 800 on the other hand showed very weak yet discernable signals in the blue and/or red channels. The photostability of the far-red dyes were also monitored (as they can be used to visualize the mitochondrial network during time lapse imaging). Under continuous imaging with four colors (405, 488, 559, 635 nm) Mitotracker Deep Red FM and Rhodamine 800 were able to maintain their fluorescence while Mitoview 633 displayed more sensitivity toward photobleaching (Fig. 3C). Mitotracker Deep Red FM therefore possesses the best spectral and photo-physical properties for live-cell imaging.

**Figure 4:**
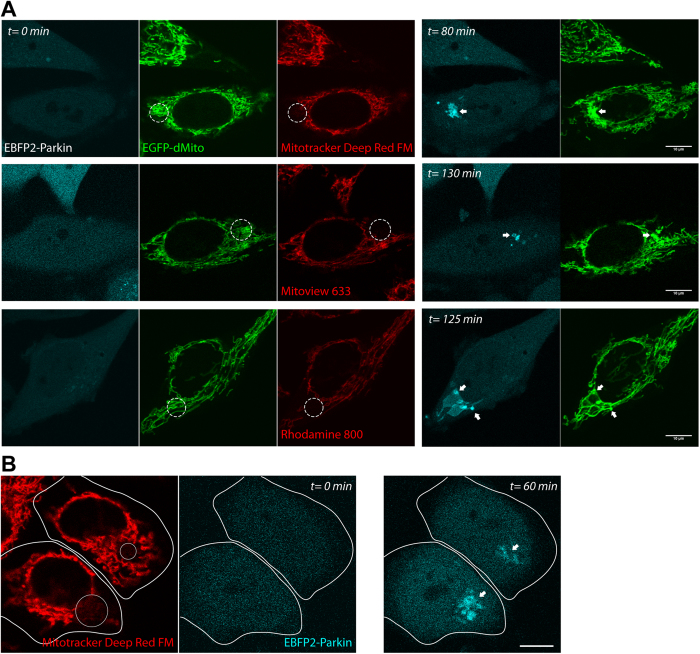
Successful induction of Parkin translocation using far-red fluorescent photosensitizers. (**A**) HeLa cells coexpressing EBFP2-LC3B, EGFP-dMito (mitochondrial-matrix targeted EGFP), and stained with far-red fluorescent photosensitizers were illuminated within the respective white dotted circular regions, leading to spatial-selective recruitment of EBFP2-Parkin onto mitochondria (white arrows). (**B**) Two HeLa cells expressing EBFP2-Parkin were stained with Mitotracker Deep Red FM, and 635 nm-illuminated within the respective white circular regions (*upper cell:* smaller area; *lower cell*: larger area). The illumination area determined the amount of cellular parkin-labeled mitochondria (white arrows). Scale bars, 10 μm.

**Figure 5:**
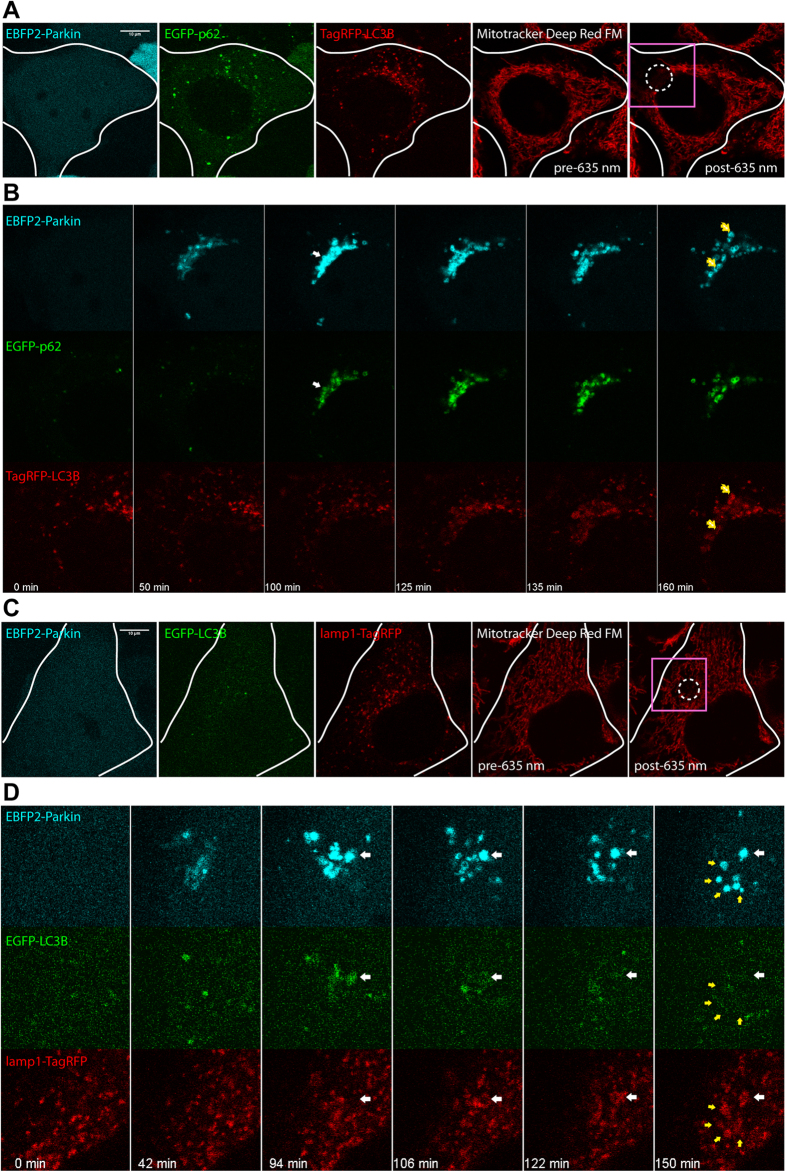
Light-activated mitophagy using far-red fluorescent photosensitizers. 635 nm illumination triggered parkin-mediated mitophagy within Mitrotracker DeepRed FM stained HeLa cells. (**A**) A HeLa cell coexpressing EBFP2-LC3B, EGFP-p62, and TagRFP-LC3B were 635 nm illuminated within the white dotted circular region. (**B**) Magnified view of the pink rectangular region in **A** 0–160 minutes post 635 nm illumination. Damaged mitochondria first become labeled with Parkin, followed by p62 (white arrow), and finally LC3 (yellow arrows). (**C**) A HeLa cell coexpressing EBFP2-Parkin, EGFP-LC3B, and lamp1-TagRFP were 635 nm illuminated within the white dotted circular region. (**D**) Magnified view of the pink rectangular region in **C** 0–150 minutes post 635 nm illumination. Parkin-labeled mitochondria eventually resided within lamp1-positive autolysosomes (yellow arrows). White arrow indicates a mitophagosome (containing Parkin-labeled mitochondria) maturing into an autolysosome (loss of EGFP signal due to low pH; gain of lamp1-TagRFP fluorescence). Scale bars, 10 μm.

Having confirmed that these far-red dyes possess favorable photo-physical properties we verified their use for inducing mitophagy. It has previously been demonstrated that targeted 635 nm illumination (sub-mW) alone does not induce Parkin-mediated mitophagy in HeLa cells^18^. In contrast, mitochondrial far-red dyes coupled with targeted 635 nm illumination led to Parkin-mediated mitophagy in HeLa cells: this is evidenced by the spatial-selective translocation of Parkin onto mitochondria (Fig. 4A), followed by the appearance of p62, LC3B, and lamp1 signals (Fig. 5). The number of mitochondria undergoing mitophagy in a HeLa cell could be controlled by the 635 nm illumination pattern (Fig. 4B). Larger irradiation areas led to the generation of higher amounts of Parkin-labeled mitochondria. These establish the three far-red dyes as adequate photosensitizers for triggering mitophagy.

### Substrate quantity affects mitophagy progression

**Figure 6:**
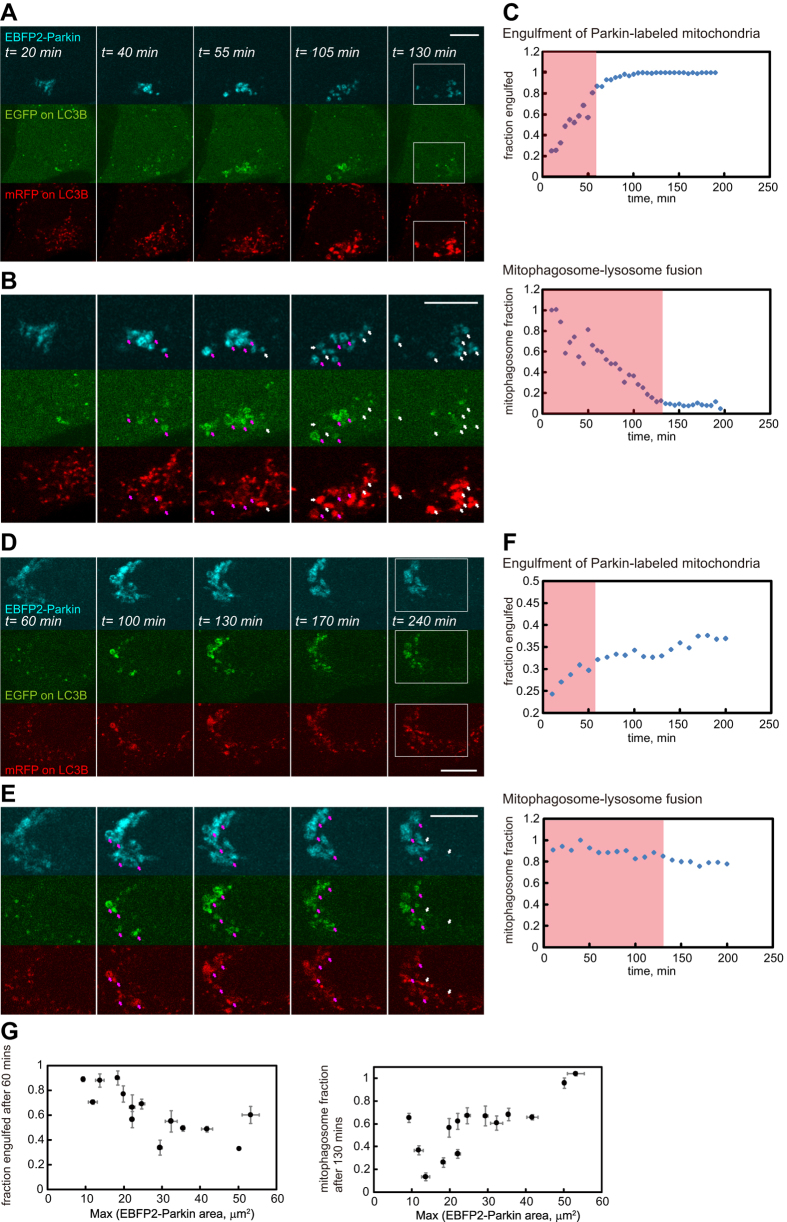
Cellular amount of Parkin-labeled mitochondria affects mitophagy progression. We photo-generated varying amounts of Parkin-labeled mitochondria (mitophagy substrate) within HeLa cells coexpressing EBFP2-Parkin and mRFP-EGFP-LC3B (Mitotracker Deep Red FM stained). Two example cells with different levels of Parkin-labeled mitochondria are shown (**A** and **D**). (**A**) A cell with lower amount of mitophagy substrate. (**B**) Magnified view of the white rectangular region in **A**. Pink arrows indicate Parkin-labeled mitochondria in autophagosomes. White arrows indicate Parkin-labeled mitochondria in autolysosomes. Note the increase of white arrows as time progresses. Parkin-labeled mitochondria became fully decorated with LC3 signals within 60 minutes in this cell. (**C**) *Top:* fraction of Parkin-labeled mitochondria engulfed into LC3B structures as a function of time for the cell in **A**. Almost all Parkin-labeled mitochondria became engulfed into autophagic structure in 60 minutes. *Bottom:* fraction of mitophagic structures yet to fuse with lysosomes as a function in time for the cell in **A**. Almost all mitophagosomes fused with lysosomes (matured) within 130 minutes. The definition of these two parameters is detailed in the Materials and Methods section. In this graph we defined *t = 0* as the instant when EBFP2-Parkin signals became detectable on impaired mitochondria. (**D**) A cell with higher amount of mitophagy substrate. (**E**) Magnified view of the white rectangular region in **D**. Pink arrows indicate Parkin-labeled mitochondria in autophagosomes. White arrows indicate Parkin-labeled mitochondria in autolysosomes. (**F**) *Top:* Fraction of Parkin-labeled mitochondria engulfed into LC3B structures as a function of time for the cell in **D**. Only ~30% of Parkin-labeled mitochondria were engulfed into autophagic structures in 60 minutes. *Bottom:* fraction of mitophagic structures yet to fuse with lysosomes as a function in time for the cell in **D**. Less than 20% of the mitophagosomes fused with lysosomes within 130 minutes. (**G**) Extent of mitophagic engulfment (*left*, at *t = 60 min*) and mitophagosome maturation (*right*, *at t = 130 min*) vs. substrate amount. Each point on the graphs represents data obtained from a single cell. The maximum detected EBFP2-Parkin area within individual image sequences was used as an indicator for the Parkin-labeled substrate amount in each cell. Scale bars, 10 μm.

**Figure 7:**
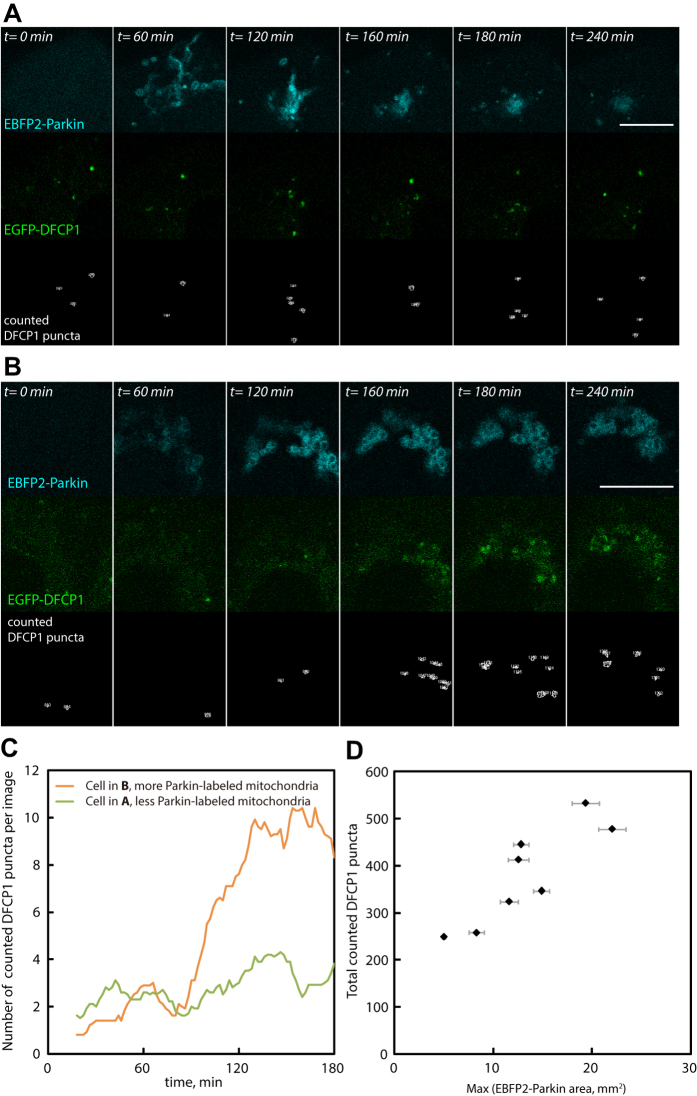
Cellular parkin-labeled mitochondria amount affects mitophagic omegasome formation. We photo-generated varying amounts of Parkin-labeled mitochondria (mitophagy substrate) within different HeLa cells (coexpressing EBFP2-Parkin, EGFP-DFCP1, and KillerRed-dMito). Here we show two example cells with different amounts of Parkin-labeled mitochondria (**A** and **B**). (**C**) Identified DFCP1 puncta number (on Parkin-labeled mitochondria) per image in the two cells in **A** and **B** at different times after mitochondrial impairment. (**D**) Total counted DFCP1 puncta on Parkin-labeled mitochondria (within four hours after EBFP2-Parkin translocation; imaging time interval, 2 min) vs. substrate amount. Each point on the graphs represents data obtained from a single cell. The maximum detected EBFP2-Parkin area within individual image sequences was used as an indicator for the Parkin-labeled substrate amount in each cell. Scale bars, 10 μm.

We utilized the newly identified far-red fluorescent photosensitizers to probe how cells cope with different extent of mitochondrial injury through mitophagy. One situation a cell may need to avoid is targeting too many mitochondria for autophagic removal at once: this could rapidly deplete intracellular membrane sources and overwhelm the lysosomal system. To investigate this we systematically photo-generated varying amounts of Parkin-labeled mitochondria in HeLa cells (converting up to ~15% of total mitochondria at once), and utilized EBFP-Parkin and mRFP-GFP-LC3B to track how mitophagy progression differed with substrate quantity^23^. The percentage of Parkin-labeled mitochondria (EBFP2) that became decorated with LC3B structures (mRFP) was used as a measure for autophagic engulfment, and the fraction of mitophagic structures that had lost their EGFP fluorescence was taken to indicate mitophagosome maturation (EBFP2-Parkin structures that displayed mRFP signals from LC3B, but without EGFP fluorescence; mitophagosome-lysosome fusion leads to the attenuation of EGFP fluorescence on LC3B). Using these two measures we found when substrate amounts were lower autophagy engulfed the entire Parkin-labeled mitochondria efficiently, and mitophagosomes matured rapidly (Fig. 6A–C). On the other hand in cells with higher quantity of Parkin-labeled mitochondria the engulfment and maturation efficiency decreased dramatically (Fig. 6D–F). The mitophagic engulfment or mitophagosome maturation efficiency strongly correlated with cellular amounts of Parkin-labeled mitochondria (Fig. 6G; Spearman’s coefficients: –0.82 [p-value < 0.005] and 0.77 [p-value < 0.005], respectively). This occurred even with the fact that cells displayed higher numbers of mitophagosome initiation sites (DFCP1 labeled omegasomes on Parkin-labeled structures^24^) when challenged with increasing substrate amounts (Fig. 7; Spearman’s coefficient: 0.90 [p-value < 0.005]).

## Discussion

Here we explored the use of Mitotracker DeepRed FM, Mitoview 633, and Rhodamine 800 as far-red fluorescent mitochondrial photosensitizers. We found that these fluorophores selectively label mitochondria in HeLa cells and display low cytotoxicity, making them compatible with long term live-cell imaging. Most importantly they are as effective as TMRE in mediating ROS production when irradiated, and therefore can be used to trigger mitophagy using light sources commonly equipped on regular fluorescence microscopes. The far-red dyes are commercially available, making them easily accessible to most research groups.

The identification of the above mentioned mitochondrial photosensitizers means that one can now activate mitophagy with red light. Even when visualization of the mitophagy substrate through fluorescently-tagged Parkin is desired, there will still be room for simultaneous use of two additional fluorescent reporters during live-cell imaging. The use of far-red photosensitizers therefore makes the light-activated scheme a much more realistic and flexible approach for investigating mitophagy.

Taking advantage of the far-red photosensitizers we investigated how cells cope with differential levels of defective mitochondria during mitophagy. We utilized three fluorophores (in EBFP2-Parkin and mRFP-EGFP-LC3B) to monitor this effect in detail, a configuration allowed through the use of far-red photosensitizers. We found that cells displayed significantly delayed autophagic engulfment and mitophagosome maturation when large quantities of mitophagic substrates are present. This may represent a protective cellular mechanism to avoid membrane and lysosome depletion during mitophagy.

## Materials and Methods

### Plasmids

KillerRed-dMito, EBFP2-LC3B, EGFP-DFCP1, EGFP-p62, lamp1-TagRFP, and EBFP2-parkin were described previously^13^,^18^,^25^. EGFP-dMito was constructed by PCR amplification of the mitochondrial targeting sequence from KillerRed-dMito, followed by its insertion into EGFP-C1. EYFP-parkin was purchased from Addgene (plasmid 23955^4^). mRFP-EGFP-LC3B was a gift from Dr. Tomatsu Yoshimori^23^.

### Cell culture conditions and transfections

HeLa cells (ATCC, CCL-2) were grown in DMEM medium (Life Technologies, #11965), supplemented with 10% FBS (Life Technologies, #10437) and 1% penicillin/streptomycin (Life Technologies, #15140), and maintained at 37 ^o^C and 5% CO_2_. Cells were transfected using Lipofectamine 2000 (Life Technologies, #11668) according to manufacturer’s recommendations.

### Photosensitizers

Mitotracker Deep Red FM (Life Technologies, #M22426), Mitoview 633 (Biotium, #70055), Rhodamine 800 (Sigma, #83701), and TMRE (Life Technologies, #T-669) were used without further purification. Photosensitizer stock solutions were prepared in DMSO (Sigma, #D2650). Absorption spectra of the photosensitizers were determined on a UV-Vis spectrophotometer (Beckman Coulter, #DU-800) in PBS buffer (pH 7.4) at room temperature within a quartz cuvette (Nova Biotech, #QS-467). Condition for labeling HeLa cell mitochondria with photosensitizers: 100 nM in complete medium at 37 ^o^C for 30 min (followed by 3X PBS wash).

### Time lapse imaging and far-red light activated mitophagy

Light-assisted parkin-mediated mitophagy was performed on Olympus FV1000 confocal microscopes (60X N.A. = 1.2 water objective). HeLa cells were maintained under 37 ^o^C and 5% CO_2_ (Tokai Hit, #MIU-IBC) in phenol-red free medium (Life technologies, #31053, containing FBS and P/S). We point-scanned 635 nm laser (100 μW) within HeLa cells using Olympus FV1000’s tornado scanning (3 μm diameter ROI, 30 seconds; multiple ROIs can be sequentially illuminated to tune the amount of mitochondria undergoing mitophagy) to locally activate mitophagy. Mitophagy was followed by imaging live cells at fixed time intervals, and a zero drift compensator module (ZDC from Olympus) was used to correct sample drift in the z-direction during live cell imaging.

### Quantifying ROS generation

#### In vitro measurements

Individual photosensitizer’s ability to generate ROS upon irradiation was quantified *in vitro* using either **(a)** N,N-Dimethyl-4-nitrosoaniline (RNO) plus imidazole (Sigma, #D172405 and #I2399)^19^ or **(b)** dichlorofluorescin (DCFH; DCFH were generated through treating 2,7-dichlorodihydrofluorescein diacetate with NaOH)^20^. In **a** singlet oxygen generated upon photosensitizer irradiation reacts with imidazole and subsequently bleaches RNO through oxidation (RNO bleaching were monitored through measuring the reduction of its absorption at 438 nm). In **b** the generated ROS reacts with DCFH to form dichlorofluorescein (DCF; DCF formation were detected through its absorption at 501 nm). Reaction mixtures containing either 7 μM photosensitizer, 75 μM RNO, and 8 mM imidazole in PBS (pH7.4) or 7 μM photosensitizer and 45 μM DCFH in PBS (pH7.4) were held in quartz cuvettes (Nova Biotech, #QS-467) mounted on a homemade irradiation system (Thorlabs, #M660L3-C1 # M565L2, and #DC2100). Samples were continuously illuminated (660 nm for Mitotracker Deep Red FM, Mitoview 633, and Rhodamine 800; 565 nm for TMRE) and assayed for singlet oxygen generation. We normalized the readouts with individual photosensitizers’ molar extinction coefficients and illumination intensities (660 nm LED power: 0.57 mW; 565 m LED power: 0.18 mW) at respective wavelengths for comparison.

#### Cellular measurements

Individual photosensitizer’s ability to generate ROS upon irradiation was also quantified directly within HeLa cell cultures. HeLa cells were co-labeled with 100 nM photosensitizers and live-cell ROS fluorescent indicators (5 μM MitoSOX Red [Invitrogen, M36008] or 20 μM DCF-DA) in cell culture medium (37 ^o^C for 30 min, followed by 3X PBS wash). 8 μm × 8 μm regions within cells were pointed scanned with 100 μW 635 nm light for 30 seconds, and the corresponding increase in mitochondrial MitoSOX or DCF fluorescence were quantified using ImageJ. We normalized the readouts with individual photosensitizers’ molar extinction coefficients and illumination intensities at respective wavelengths for comparison.

### Photostability test

HeLa cells stained with far-red photosensitizers were continuously imaged with 635 nm alone or simultaneously with four colors (405, 488, 559, 635 nm; excitation intensity: 200 nW-1 μW). The photosensitizers’ fluorescence intensity where monitored over time to evaluate their relative photostability during live-cell imaging.

### Mitophagy flux analysis

#### RFP-GFP-LC3

HeLa cells coexpressing EBFP2-parkin and mRFP-GFP-LC3 were stained with Mitotracker DeepRed FM for light-activated mitophagy. Cells were then imaged at 2–5 min intervals for up to 4 hours for mitophagy flux analysis (in ImageJ). Each channel (Blue: EBFP2; Green: EGFP, Red: mRFP) was first smoothed and intensity-thresholded to remove cytosolic signals. The EBFP2-parkin images were used to define ROIs containing parkin-labeled mitochondria for downstream analysis of two parameters. **Parameter 1** (indicator for Parkin-labeled mitochondria engulfment): fraction of parkin-labeled mitochondria covered by autophagic structures. This was calculated by counting the fraction of pixels within the ROIs that have mRFP signals above the intensity threshold. This parameter measures how efficiently autophagy engulfs damaged mitochondria. **Parameter 2** (indicator for mitophagosome maturation): fraction of mitochondria-containing autophagic structures that remained as autophagosomes. This was calculated by taking the total intensity ratio between EGFP and mRFP within ROIs defined by BFFP2-Parkin signals (only EGFP and mRFP signals that are above the intensity threshold were counted). This parameter measures the mitophagosome maturation rate. When calculating both parameters 1 and 2 time zero (*e.g.* in Fig. 6C,F) indicates the very instant when EBFP2-Parkin signals become detectable on damaged mitochondria.

#### EGFP-DFCP1

HeLa cells coexpressing EBFP2-parkin, EGFP-DFCP1, and KillerRed-dMito were light-activated to undergo mitophagy through 559 nm laser illumination as previously described^13^. Cells were then imaged at 2 min intervals. EGFP-DFCP1 spots within each image was identified and counted using the “Analyze Particles” tool in ImageJ.

## Additional Information

**How to cite this article**: Hsieh, C.-W. *et al.* Triggering mitophagy with far-red fluorescent photosensitizers. *Sci. Rep.* **5**, 10376; doi: 10.1038/srep10376 (2015).
