# Corrigendum: Triggering mitophagy with far-red fluorescent photosensitizers

**DOI:** 10.1038/srep27167

**Published:** 2016-06-10

**Authors:** Cheng-Wei Hsieh, Chih-Hang Chu, Hsien-Ming Lee, Wei Yuan Yang

Scientific Reports 5: Article number: 10376; 10.1038/srep10376 published online: 05292015; updated: 06102016

In the original version of this Article, an additional affiliation for Cheng-Wei Hsieh was omitted. The correct affiliation is listed below:

Department of Chemistry, National Tsing-Hua University, Hsinchu 30013, Taiwan.

In addition, Affiliations 1 and 2 were incomplete. The correct affiliations are listed below.

Affiliation 1:

Chemical Biology and Molecular Biophysics Program, Taiwan International Graduate Program, Academia Sinica, Taipei 115, Taiwan.

Affiliation 2:

Institute of Biological Chemistry, Academia Sinica, Taipei 115, Taiwan.

These errors have now been corrected in the PDF and HTML versions of the Article.

